# Supplementary material for: DNA damage induced by HIV-1 Vpr triggers epigenetic remodeling and transcriptional programs to enhance virus transcription and latency reactivation
Source: PLoS Biol. 2026 Feb 2;24(2):e3003621. doi: 10.1371/journal.pbio.3003621 (PMC12875578; doi:10.1371/journal.pbio.3003621)

REPRESENTATIVE FLOW CYTOMETRY GATING STRATEGIES

FIGURES 1D, S2B

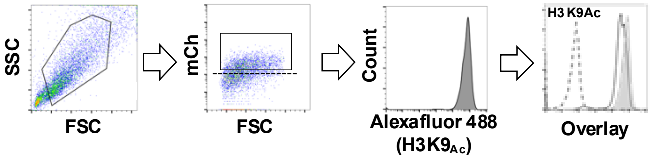

FIGURE 2D

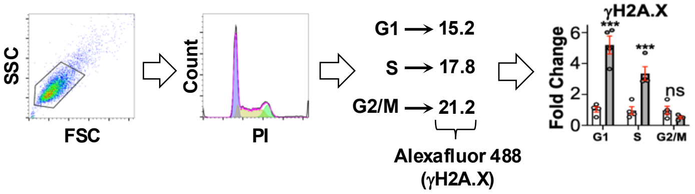

FIGURES 7A, S4A

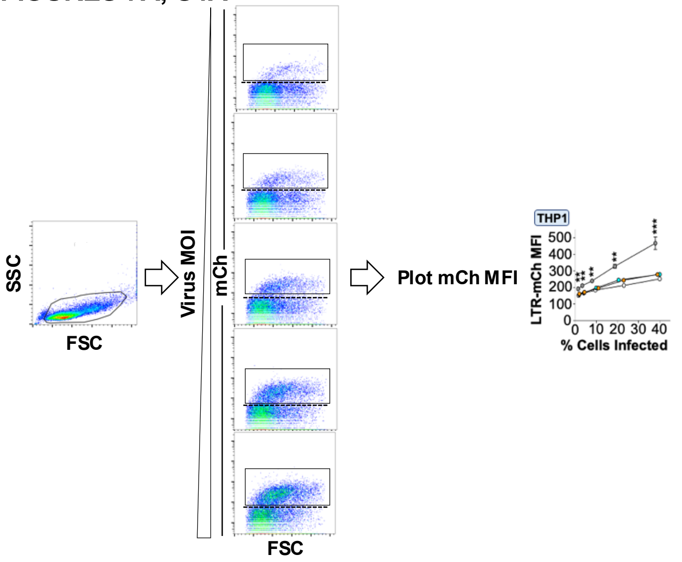

FIGURE 7B, 7C, 8G

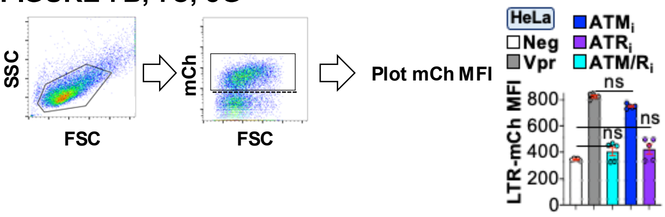

FIGURE 7D

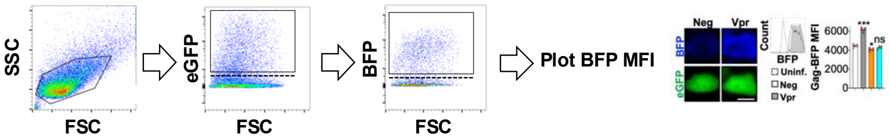

FIGURES 7F, 7G and 7I

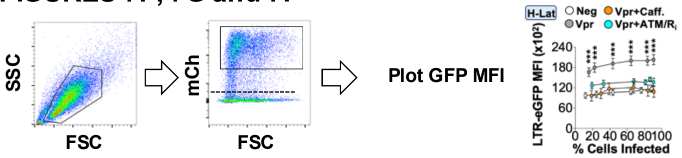

FIGURE 7H

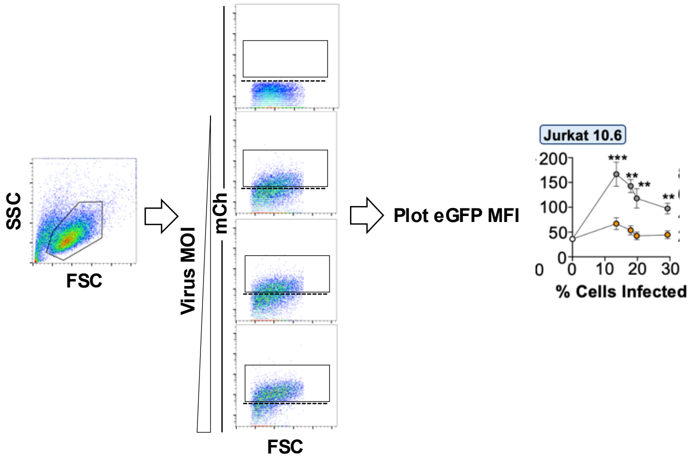

Supplement: S2 Data — Representative gating strategies are depicted for the indicated experiments. Accompanying figure panels for which the gating strategy is relevant are listed above each workflow. (PDF) [file pbio.3003621.s002.pdf]
