# Supplementary figures and images for: DNA damage induced by HIV-1 Vpr triggers epigenetic remodeling and transcriptional programs to enhance virus transcription and latency reactivation
Source: PLoS Biol. 2026 Feb 2;24(2):e3003621. doi: 10.1371/journal.pbio.3003621 (PMC12875578; doi:10.1371/journal.pbio.3003621)

FIGURE 1

A

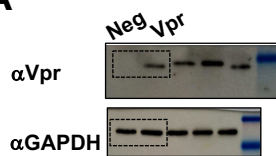

C

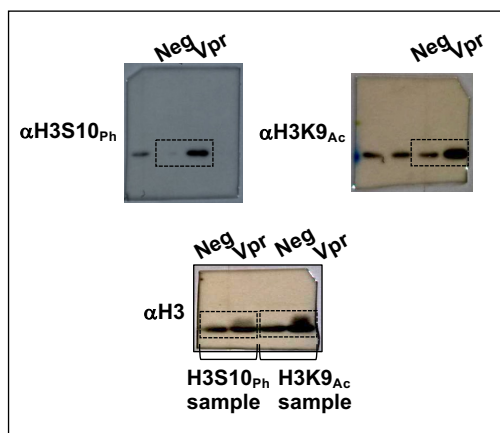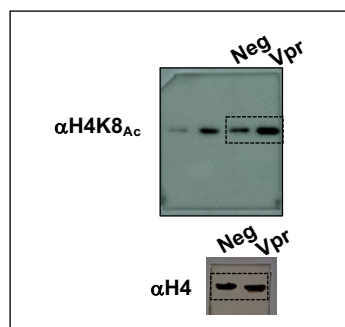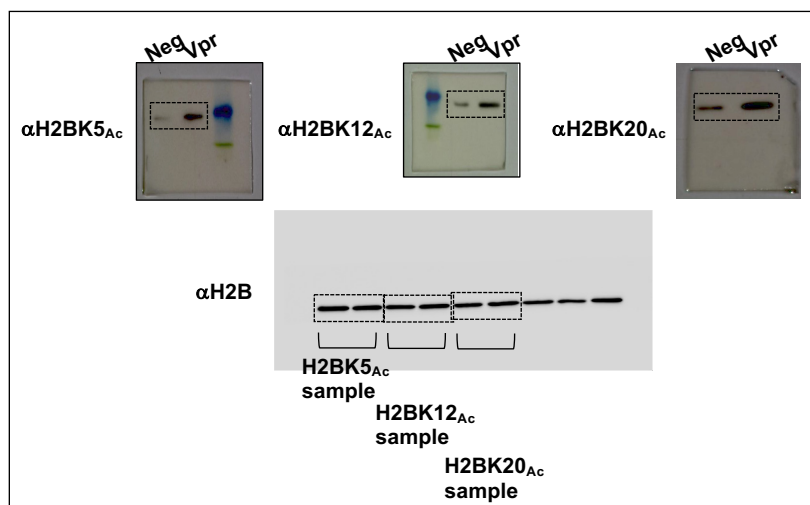

FIGURE 4

D

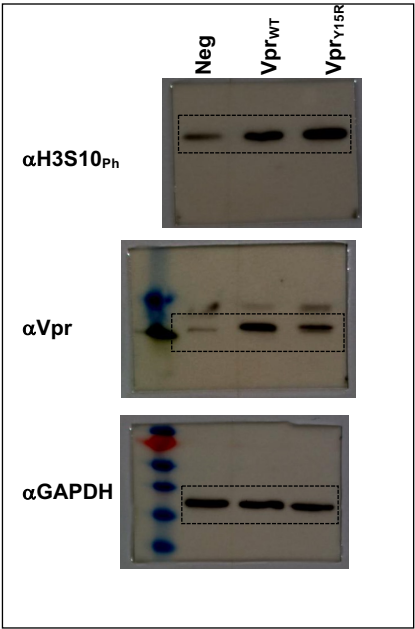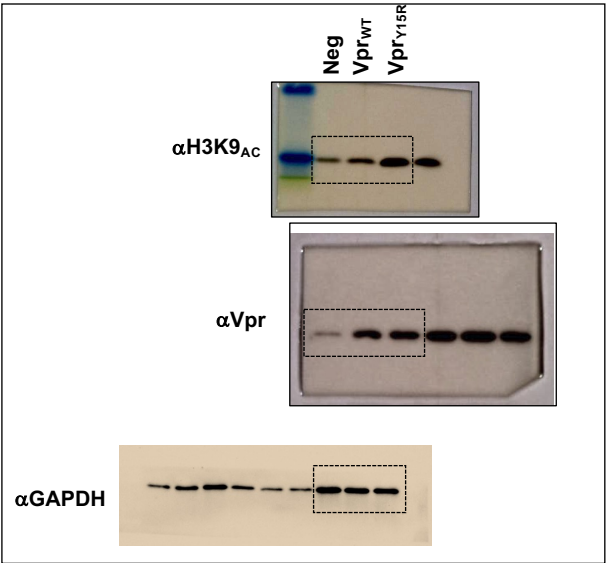

FIGURE S2

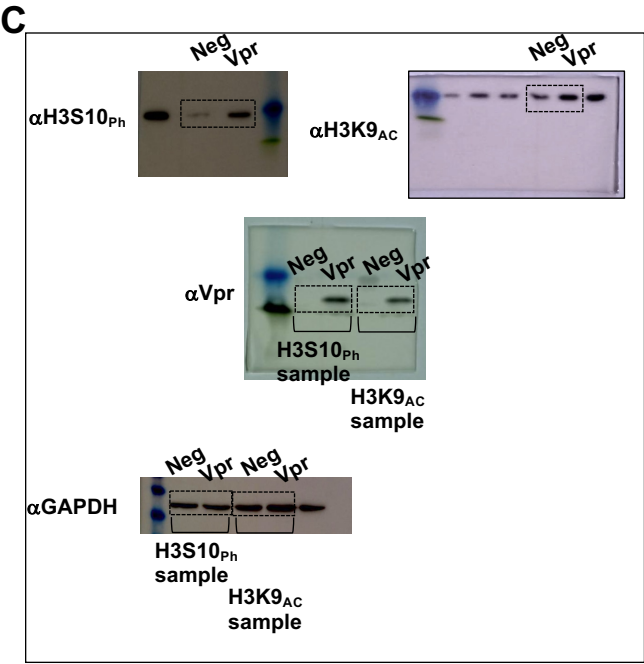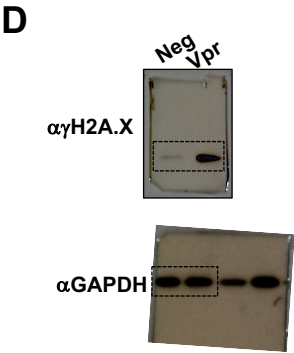

FIGURE S3

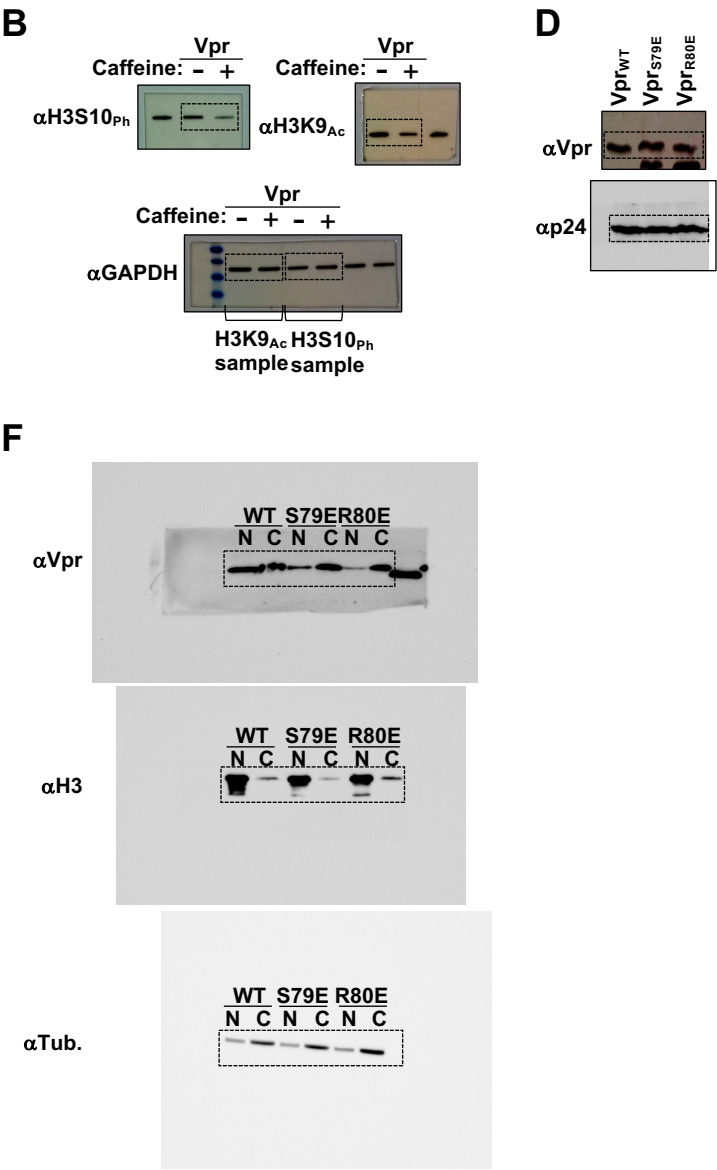

FIGURE S4

H

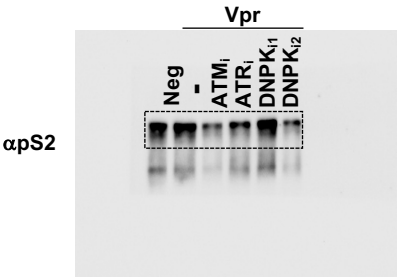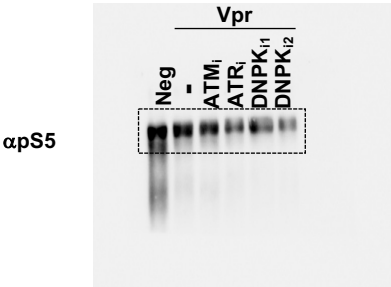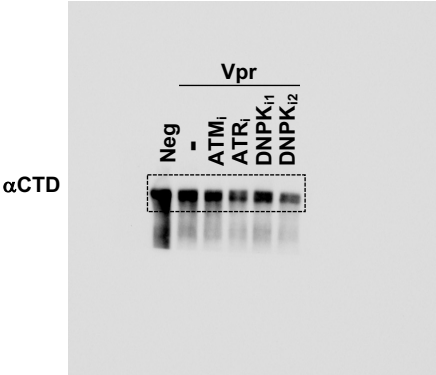

Supplement: S1 Raw Images — Original immunoblot images are depicted for the indicated figure panel with the dashed box representing the image used in the manuscript. (PDF) [file pbio.3003621.s003.pdf]

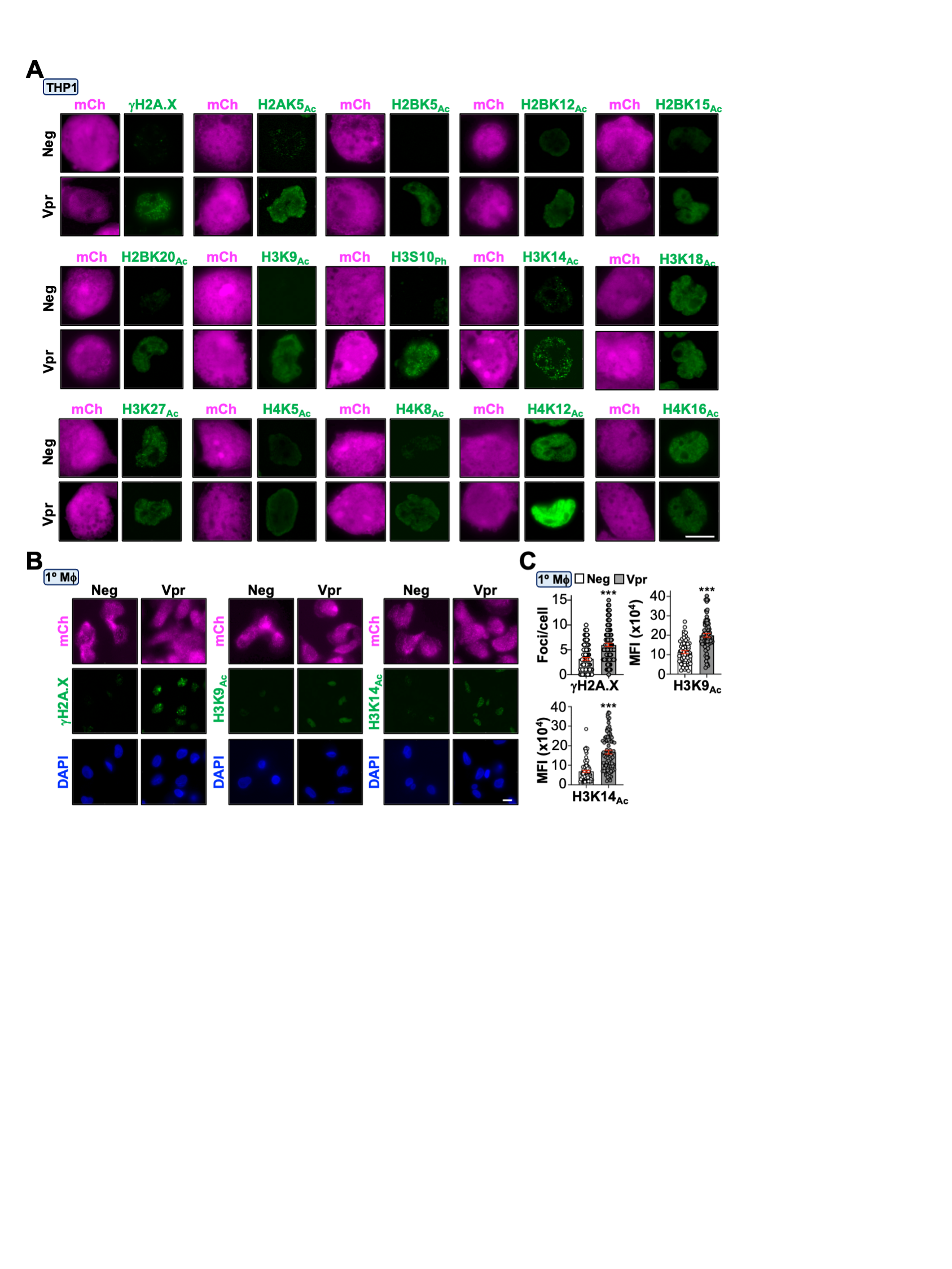

Supplement: S1 Fig — (A) Representative fluorescence microscopy images of single nuclei from differentiated THP1 cells infected with control or Vpr-expressing viruses. Indicated histone marks are visualized using specific anti-acetylation or -phosphorylation antibodies. (B) Representative fluorescence microscopy images of histone marks in primary MDM cells infected with indicated viruses. (C) Quantification of histone marks following infection of primary MDM cells with VprWT or control viruses (n = 75 cells). The data underlying this Figure can be found in S1 Data. (TIFF) [file pbio.3003621.s004.tiff]

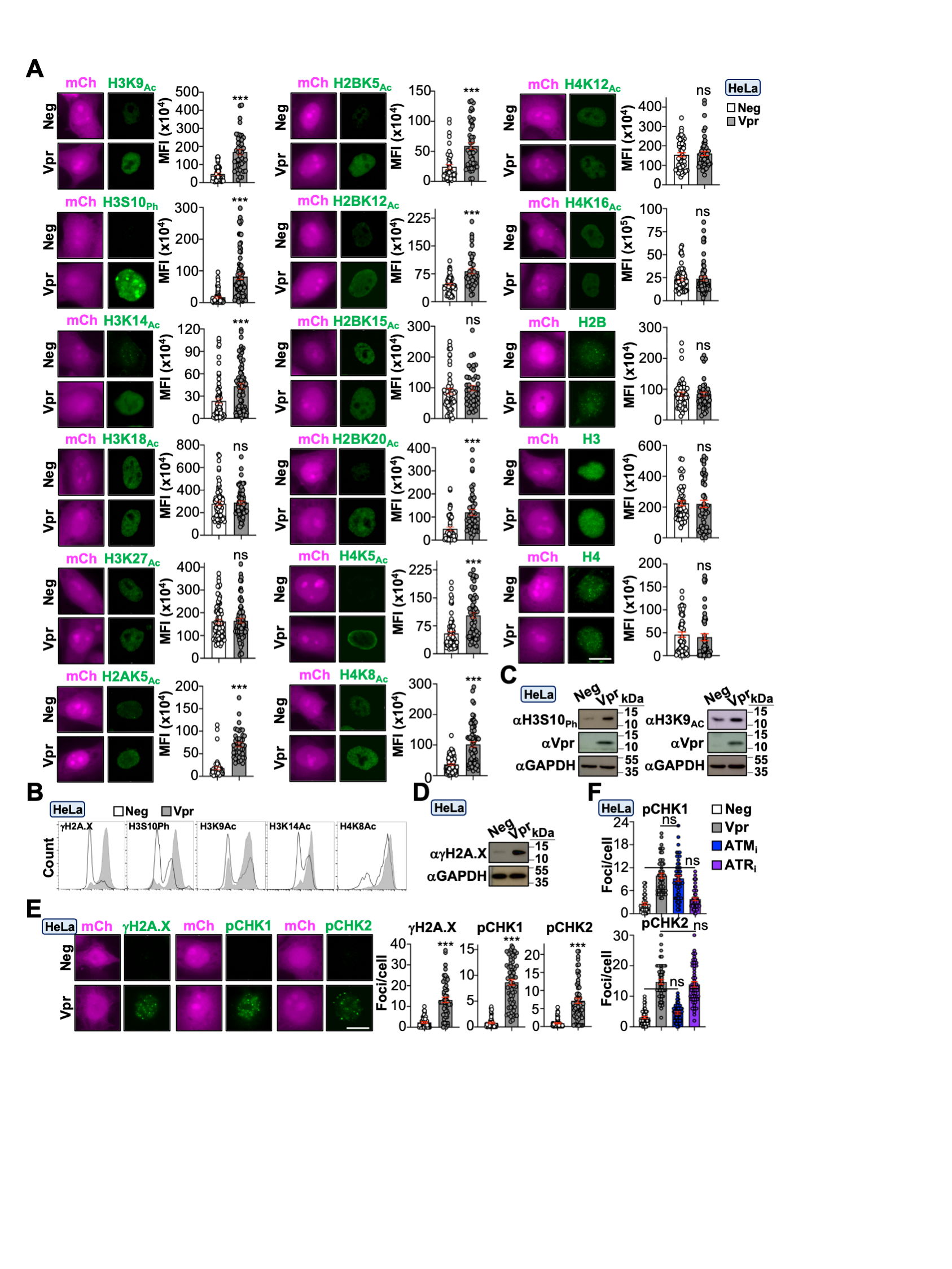

Supplement: S2 Fig — (A) Representative fluorescence microscopy images and quantification of histone marks in HeLa cells infected with indicated viruses (n = 50 cells). Analyses performed using a student t test; ns, not significant; *** p < 0.001. The data underlying this Figure can be found in S1 Data. (B) Flow cytometric analysis of DDR activation and histone marks in HeLa cells infected with indicated viruses. Representative gating strategies are depicted in S2 Data and raw FSC files can be found in the Figshare Data repository (https://doi.org/10.6084/m9.figshare.c.8239897). (C) Immunoblot analysis of histone marks in HeLa cells infected with indicated viruses. The unmodified images underlying this Figure can be found in S1 Raw Images. (D, E) Immunoblot analysis (D) and representative fluorescence microscopy images and quantification (E) of DDR activation in HeLa cells infected with indicated viruses (n = 50 cells). Analyses performed using a student t test; *** P < 0.001. The data underlying this Figure can be found in S1 Data. The unmodified images underlying this Figure can be found in S1 Raw Images. (F) Quantification of DDR activation following infection of HeLa cells with the indicated virus in the presence or absence of vehicle, 10 nM ATMi, or 10 mM ATRi (n = 50 cells). Analyses performed using a one-way ANOVA; ns, not significant; *** p < 0.001. The data underlying this Figure can be found in S1 Data. (TIFF) [file pbio.3003621.s005.tiff]

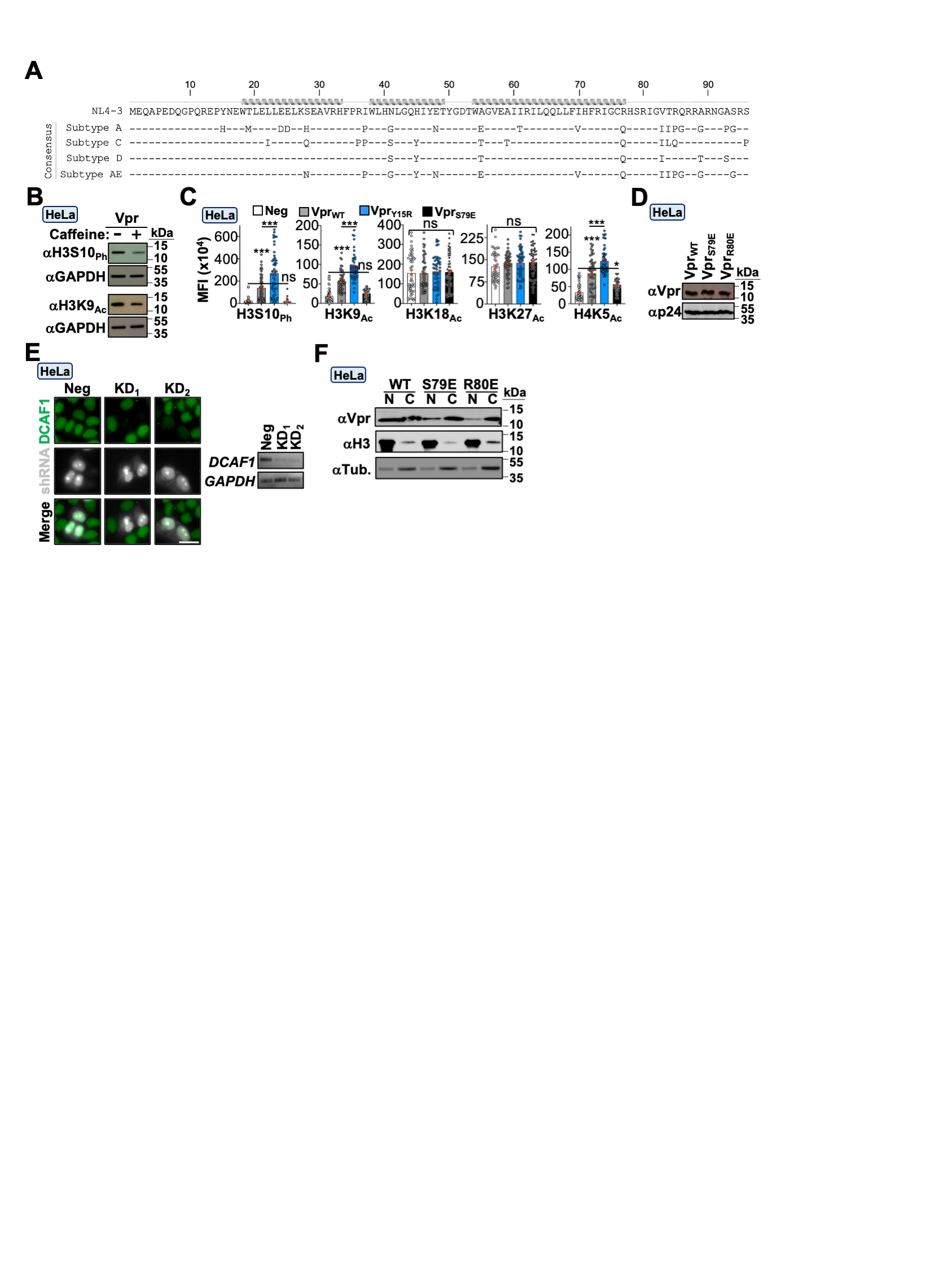

Supplement: S3 Fig — (A) Amino acid alignment of HIVNL4-3 and consensus subtype Vpr sequences. (B) Immunoblot analysis of indicated histone marks of VprWT infected HeLa cells in the presence and absence of 3 mM caffeine at 48 hours post-infection. (C) Quantification of histone marks following infection of HeLa cells with VprWT or mutant viruses (n = 50 cells). Analyses performed using a one-way ANOVA; ns, not significant; * p < 0.05; *** p < 0.001. The data underlying this Figure can be found in S1 Data. (D) Immunoblot analysis of Vpr protein expression in HeLa cells infected with the indicated viruses 48 hours post-infection. The unmodified images underlying this Figure can be found in S1 Raw Images. (E) Representative fluorescence microscopy images and RT-PCR analysis of DCAF1 expression in HeLa cells transfected with DCAF1 shRNAs. (F) Immunoblot analysis of fractionated HeLa cell lysates infected with indicated viruses 48 hours post-infection. The unmodified images underlying this Figure can be found in S1 Raw Images. (TIFF) [file pbio.3003621.s006.tiff]

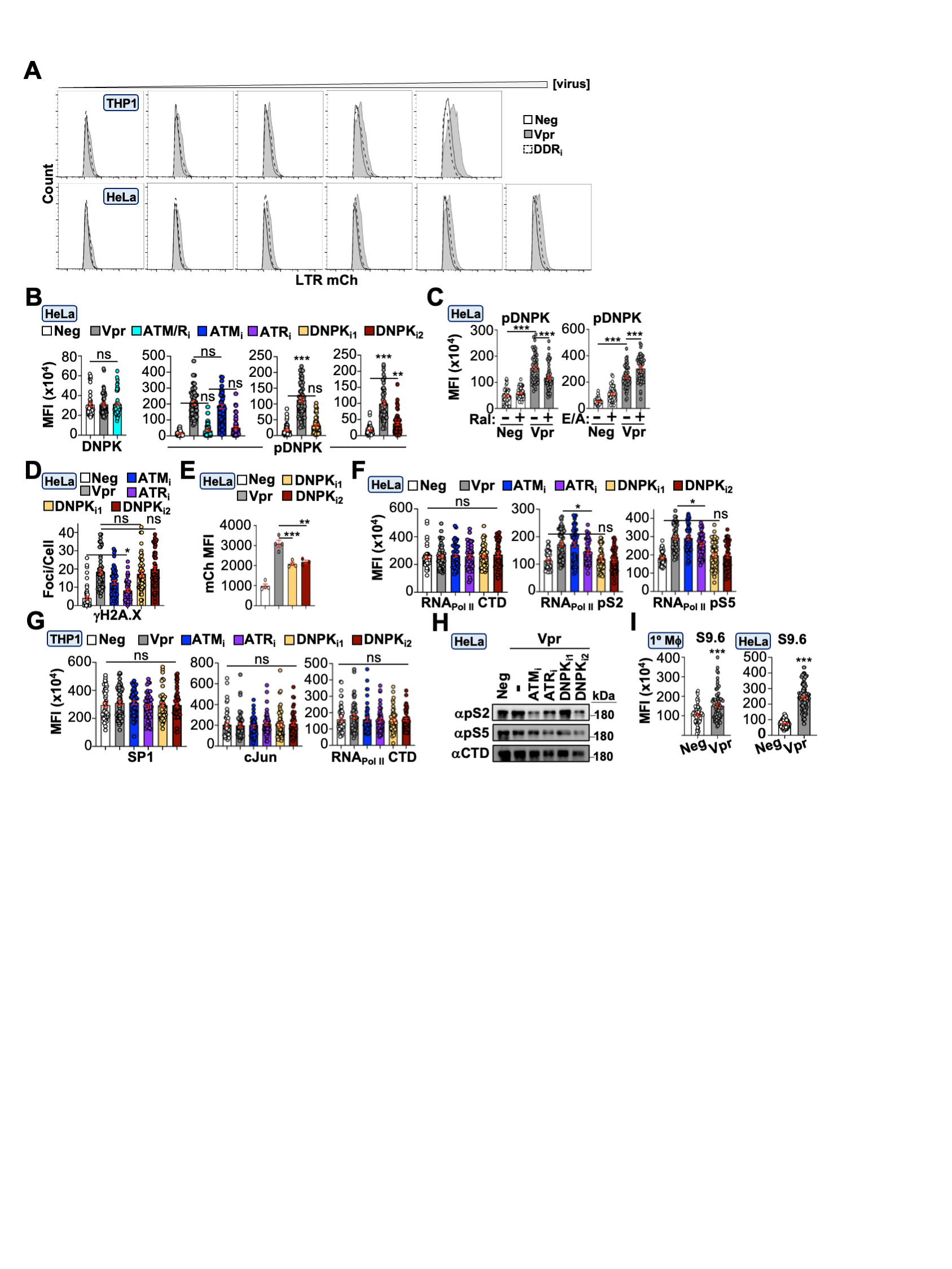

Supplement: S4 Fig — (A) Flow cytometric analysis of HIV LTR activity in THP1 (top) and HeLa (bottom) cells infected with indicated viruses in the presence or absence of DDR inhibitors. Representative gating strategies are depicted in S2 Data and raw FSC files can be found in the Figshare Data repository (https://doi.org/10.6084/m9.figshare.c.8239897). (B) Quantification of total DNA-PK (DNPK) and phosphorylated DNA-PK (pDNPK) in HeLa cells infected with the indicated viruses in the presence or absence of ATM, ATR, and DNA-PK inhibitors (n = 50 cells). Analyses performed using a one-way ANOVA; ns, not significant; *** p < 0.001; ** p < 0.01. The data underlying this Figure can be found in S1 Data. (C) Quantification of DNPK activation in HeLa cells infected with VprWT or control viruses pre-treated with raltegravir or combination zidovudine/etravirine (n = 50 cells). Analyses performed using a one-way ANOVA; ns, not significant; *** p < 0.001. The data underlying this Figure can be found in S1 Data. (D) Quantification of gH2A.X foci in HeLa cells infected with the indicated viruses in the presence or absence of ATM, ATR, or DNA-PK inhibition (n = 50 cells). Analyses performed using a one-way ANOVA. ns, not significant. The data underlying this Figure can be found in S1 Data. (E) Quantification of mCh MFI of HeLa cells infected with VprWT or control viruses in the presence or absence of DNA-PK inhibition (n = 4 experiments). Analyses performed using a one-way ANOVA. *** p < 0.001; ** p < 0.01. The data underlying this Figure can be found in S1 Data. (F) Quantification of total RNA polymerase II (RNAPol) CTD (left), phosphorylated Ser2 (middle), and phosphorylated Ser5 (right) in HeLa cells infected with VprWT or control viruses in the presence or absence of ATM, ATR, or DNA-PK inhibition (n = 50 cells). Analyses performed using a one-way ANOVA; ns, not significant; * p < 0.05. The data underlying this Figure can be found in S1 Data. (G) Quantification of total SP1 (left), cJun (middle), [file pbio.3003621.s007.tiff]
