# Supplementary material for: DNA damage induced by HIV-1 Vpr triggers epigenetic remodeling and transcriptional programs to enhance virus transcription and latency reactivation
Source: PLoS Biol. 2026 Feb 2;24(2):e3003621. doi: 10.1371/journal.pbio.3003621 (PMC12875578; doi:10.1371/journal.pbio.3003621)
Supplement: Attachment — Submitted filename: Response_to_Reviewers_v1.docx [file pbio.3003621.s010.docx]

We thank the expert reviewers for their time and constructive comments and suggestions. Our point-by-point responses are shown below in blue.

**Reviewer #1**:

Saladino et al. test the role of virion-associated and de novo Vpr in the induction of DDR responses, looking at epigenetic remodeling and activation of transcript by the LTR promoter of HIV-1 in acute infection and reactivation from latency. They find that Vpr exists as multimers in the nucleus and monomeric in the cytoplasm and that Vpr increases transcription from the HIV-1 LTR. The paper reports a substantial body of research that addresses the still enigmatic function of the HIV-1 Vpr accessory protein. The experiments are carefully done with required controls and reference citations are accurate. The authors have developed some valuable tools in their studies, including the reporter viruses, Vpr mutants and split Venus fusion proteins. The question of Vpr function has become quite complex, comprehensible only to those working directly on it. The studies are sophisticated, but it will be hard for those outside the Vpr field to understand the story.

How does Vpr induce DDR? Presumably, it does not cleave the DNA. Is it the cell cycle arrest that does it? The authors should explain.

Response: This is an excellent question that has not been fully fleshed out experimentally. Using a variety of techniques and experimental systems, multiple groups have demonstrated that Vpr associates with nucleic acids and chromatin *in vitro* and *in cellulo*, which leads to the activation of ATR-mediated signaling, alterations to chromatin structure, and chromosome segregation [PMIDs: 9611258, 10848983, 21875947, 20824083, 16306615]. These studies speculate that this direct binding mechanism is what leads to DNA damage, independent of cell cycle arrest. However, it is also worth noting that Vpr induces the depletion of roughly half a dozen DNA repair proteins. Thus, it is likely that these two Vpr activities combine to induce global DNA damage and DDR signaling. We have added this information to the **Discussion**.

They show that the DDR is mediated by both DCAF-dependent and independent mechanisms. How does Vpr mediate these two functions?

Response: Another excellent question that has yet to be resolved experimentally. We speculate that the regulatory switch controlling these Vpr functions may involve phosphorylation of serine residues at the C-terminal tail. Previous studies have determined that protein kinase A can phosphorylate Vpr at S79, S94, and S96 [PMIDs: 20392842, 11860675, 10864665]. Moreover, Vpr has also been shown to engage 14-3-3 proteins, which specifically bind to phosphorylated proteins [PMIDs: 16968670, 15708996]. We speculate that the Vpr S79E mutant loses the ability to induce DDR signaling and nuclear retention due to mimicking constitutive phosphorylation. As noted below by Reviewer #2, S79A has been shown to induce equivalent levels of H2A.X foci as compared to wild-type but loses the ability to induce G2/M cell cycle arrest, supporting the possibility that phosphorylation of the C-terminal tail may regulate distinct functions. We have included this possibility in the **Discussion** as follows, “*Interestingly, several studies have demonstrated that Vpr is phosphorylated at residues S79, S94, and S96 [93-95] and can engage 14-3-3 proteins, which specifically bind phosphorylated targets [96, 97]. These observations raise the possibility that phosphorylation of the C-terminal tail may regulate discrete Vpr functions. In support of this idea, Vpr S79A has been shown to induce similar levels of gH2A.X foci compared to wild-type Vpr but loses the ability to induce G2/M cell cycle arrest [17, 93].*”

.

While there is a lot of data presented, it is important that it be easily intelligible to the reader. This should be improved throughout. The figures are tiny and the panels are squashed together, presumably to save space. They need to be made more easily visible.

Response: We fully appreciate this point and have made the figure panels more interpretable by shifting some data to the supplemental figures to allow for resizing, enlarging figure panels to make the data more easily accessible, and have broken up large figures into multiple figures (*ex.,* **Figure 2** is now **Figure 2** and **3**).

In addition, the figure legends do not adequately describe the experiments. It should not be necessary to consult M&Ms to understand the experiments.

Response: We apologize for the brevity and lack of details in the figure legends. We have expanded the legends to provide more details regarding the experimental designs, treatment conditions, *etc*.

Much of the data consists of histograms. Each bar shows a dot for each cell. Because of the large number of dots, the bars are difficult to discern. Why not remove the dots and substitute for error bars? Also, some of the labeling is difficult to understand, as detailed below.

Response: These are excellent points that we fully appreciate; however, for presenting data ***PLoS Biology* submission guidelines** state that *“…data is presented in such a way that the reader can evaluate the distribution of the individual data points. Scatterplots, boxplots, and histograms are more informative than bar or line charts.”* Thus, we felt compelled to display our data in the form of scatterplots. We have updated the figure panel labeling to provide more clarity in response to each point below.

**Specific points**

1.      Fig1B. What do the greyed-out boxes represent? Are the genes deleted or just point-mutated?

Response: We apologize for not clearly articulating the provirus composition in the text. The control virus retains Vif and Vpr open-reading frames, but we have incorporated mutations that render these genes non-functional. The *Vpu* gene has been deleted and *Env* has a partial deletion that retains the rev-response element but loses the ability for spread infection. For Vif, we have introduced mutations that retain APOBEC3 counteraction but lose the ability to antagonize a host phosphatase complex involved in DNA repair. For Vpr, we have incorporated two stop codons at amino acid positions 23 and 24. This information has been added to the **Experimental Procedures** section of the manuscript.

2.      Fig. 1 C. The histogram shows foci/cell or MFI. This was done using an EVOS 5000 imaging system. Some statement needs to be made of how the measurements were made. Does the microscope count the foci? Does it determine MFI/cell? The measurements were made presumably just of the mCherry+ cells. Is that correct? This needs to be stated in the figure legend.

Response: The reviewer is correct that only mCherry positive cells were considered for analysis. We have added the following text to the **Figure Legend** describing **Figure 1B** (originally **Figure 1C**), “*For quantification of immunofluorescence microscopy images, only mCherry positive cells were considered for analysis. Images were obtained using an EVOS M5000 microscope and foci or mean-fluorescence were quantified using Image J software. For foci quantification, we used the “find maxima” processing tool that quantifies foci above a preset threshold.*” This information is also detailed in the experimental procedures section titled “Experimental replicates and statistical analyses.”

3.      Fig. 1D. the images are too hard to see and there is little explanation of what they are. This needs to be improved.

Response: We apologize for the sizing and description of these images. The images have been incorporated into the supplemental material to allow for larger scaling and a more thorough description. We have increased the sizes of the images and have added the following text to the accompanying **Figure Legend**, “*Representative fluorescence microscopy images of single nuclei from differentiated THP1 cells infected with control or Vpr expressing viruses. Indicated histone marks are visualized using specific anti-acetylation or -phosphorylation antibodies*.”

4.      Fig. 2F. These images are too dark to see. This needs to be improved.

Response: We apologize for the lack of visibility. We have adjusted the exposure of these images to better visualize mCherry positive cells with and without ART treatment.

5.      Fig. 2E. The viruses are mCherry and Vpr. This nomenclature is confusing. Don't both viruses have mCherry? Does the mCherry have a Vpr mutation? Labeling needs to be clarified to increase readability. Same for the other figures.

Response: We apologize for the confusion. The reviewer is correct, both viruses express an mCherry reporter. As detailed in response point #1 above, we have engineered the control virus to encode two stop codons at amino acid positions 23 and 24 in the Vpr open-reading frame. We now label the control virus “neg” in the figures and in the text.

6.      Fig. 3. This is a vast array of Vpr point mutants. Many Vpr point mutants are known to be unstable. A western blot is required to show the relative stability of all of the mutants.

Response: We fully appreciate this point; however, our experimental goal was to identify and leverage Vpr loss-of-function mutants in subsequent experiments to correlate DDR responses with epigenetic remodeling and HIV-1 promoter activation. We include immunoblots comparing the relative abundance of wild-type Vpr to key loss-of-function (S79E, R80E) and hyperactive (Y15R) mutants in **Figures 4D**, **S3C**, and **S3F**. Moreover, eGFP-tagged Vpr loss-of-function variants in **Figures 6A** and **6B** exhibit similar expression levels compared to wild-type Vpr. Together, these observations demonstrate that functionally relevant Vpr mutants characterized herein do not exhibit expression differences compared to wild-type. While other mutations that exhibit comparable activation of DDR responses relative to wild-type may be expressed at differing levels, characterizing those functional differences is outside the scope of the current study.

7.      Fig. 5E. The split venus experiment is good but the authors conclude that Vpr exists in 2 separate pools; one in the nucleus and one in the cytoplasm. The pool in the nucleus is clearly correct but the pool in the cytoplasm doesn't agree with the well-established finding that Vpr is a nuclear protein by immunofluorescence. How would it remain monomeric in the cytoplasm anyway?

Response: Previous studies have suggested that Vpr undergoes constitutive nucleocytoplasmic shuttling and is partially relocalized to the cytoplasm following DCAF engagement [PMIDs: 11152524, 11223251]. Moreover, Vpr is abundantly packaged into nascent virions through interactions with the Gag p6 domain, which can only occur if some proportion of Vpr localizes to the cytoplasm during the infection cycle. Additionally, as mentioned above Vpr is known to be phosphorylated and interact with 14-3-3 proteins, which have been shown to sequester some host cell proteins to the cytoplasm. Thus, it is possible that sequestration of Vpr to the cytoplasm utilizes some combination of DCAF and/or 14-3-3 engagement which would also stabilize Vpr in a monomeric form. Future dedicated studies will be necessary for testing this model and determining how it impacts Vpr nuclear functions and incorporation into nascent virions.

8.      Line 168. Change text to read "It is well established"

Response: The text has been corrected

9.      Line 183. Change text to induction of "H2A/.X foci".

Response: The text has been corrected

**Reviewer #2:**

Saladino et al examine whether VPR alters the epigenomic landscape to promote increased HIV promoter activity. Here they present an extensive characterization of the changes to 17 histone marks in response to VPR+/- viruses in both HeLa and Thp1 cells. While they use microscopy as the primary assay, validation is also performed by western blot for key marks to confirm these changes via an alternate assay. Highly significant changes are also validated in primary macrophages. The authors use DDR pathway inhibitors to validate changes are linked to DNA damage induction and further perform a comprehensive mutational analysis of VPR to identify key interaction surfaces required for this activity. The authors examine the role of DCAF1 and DNA binding/nuclear retention in DDR in the generated mutants. Finally, the authors examine HIV promoter activity in an active infection model and latency models in response to VPR mutants and +/- DDR pathway inhibitors, demonstrating WT VPR does contribute to improved LTR activation during primary infection and reactivation from latency. The linkage of latency reversal to VPR is especially relevant and correlates with the known activity of HDACs as LRAs and recent work demonstrating HDACs can prevent latency establishment. The increase in acetylation induced by both VPR and HDACs are likely both remodeling chromatin genome wide and at the LTR to promote activation, a link the authors do note in the discussion.  This is a highly relevant paper that sheds light on the mechanisms of an enigmatic HIV protein. The work is well controlled, uses multiple assays and cell types for confirmation of observations, and is well presented in a logical flow.

Minor
1)      Check citation 37 on page 4, related to DDR-induced histone PTMs associated with increased HIV transcription - this doesn't seem to fit.

Response: Thank you for catching this. This citation has been removed

2)      Figure 2E - The legend does not mention primary macrophage data that is present, only references HeLa cells.  It was also difficult to interpret that the histone marks were assessed by flow instead of microscopy.  Would also recommend this be clarified in the text or legend.

Response: Thank you for catching this. The use of MDMs has been added to the Figure Legend (now **Figure 2D**) and we have clarified the methodology in both the **Figure Legend** and in the main text.

3)      Figure 2F - pCHK1/2 analysis is included here but the link between pCHK1/2 and DDR is not mentioned in the main text until Figure 3.  Recommend moving this to introduce this earlier for those not immediately familiar with all DDR pathways.

Response: We appreciate this point and have included the following information immediately prior to discussing the pCHK1/2 analysis, “*The hierarchy of DDR signaling requires the phosphorylation of H2A.X to promote the activation of ATM and ATR repair kinases, which then directly activate effector kinases CHK2 and CHK1, respectively.*”

4)      Prior work has shown the S79A mutation shows similar levels of H2A.x foci, however the S79E mutation presented here dramatically lowered these numbers.  It would be nice to a comment about the differences between these mutations and potential hypothesis as to the different phenotypes in the discussion.

Response: We speculate that the observed difference in H2A.X focus formation between the two mutants is due to the nature of the amino acid substitution. Previous studies have determined that protein kinase A can phosphorylate Vpr at S79, S94, and S96 [PMIDs: 20392842, 11860675, 10864665]. Moreover, Vpr has also been shown to engage 14-3-3 proteins, which specifically bind to phosphorylated proteins [PMIDs: 16968670, 15708996]. We speculate that the Vpr S79E mutant loses the ability to induce DDR signaling and loses nuclear retention due to mimicking constitutive phosphorylation, which would also be consistent with the S79A mutation being able to induce equivalent levels of H2A.X foci as compared to wild-type. We have included this possibility in the **Discussion** section as follows, “*Interestingly, several studies have demonstrated that Vpr is phosphorylated at residues S79, S94, and S96 [93-95] and can engage 14-3-3 proteins, which specifically bind phosphorylated targets [96, 97]. These observations raise the possibility that phosphorylation of the C-terminal tail may regulate discrete Vpr functions. In support of this idea, Vpr S79A has been shown to induce similar levels of gH2A.X foci compared to wild-type Vpr but loses the ability to induce G2/M cell cycle arrest [17, 93].*”
